# Diet and Nutrition for Body Weight Management

**DOI:** 10.1155/2019/6798096

**Published:** 2019-03-11

**Authors:** Assim A. Alfadda, Reem M. Sallam, Jiyoung Park

**Affiliations:** ^1^Obesity Research Center, College of Medicine, King Saud University, P.O. Box 2925(98), Riyadh 11461, Saudi Arabia; ^2^Department of Medicine, College of Medicine, King Saud University, P.O. Box 2925(38), Riyadh 11461, Saudi Arabia; ^3^Department of Medical Biochemistry, Faculty of Medicine, Ain Shams University, Cairo, Egypt; ^4^School of Life Sciences, Ulsan National Institute of Science and Technology, Ulsan, Republic of Korea

Body weight is regulated by several mechanisms including genetic, physiologic, and behavioural factors. In obesity, an imbalance occurs between food intake and energy expenditure, which leads to an excess fat accumulation and negative health consequences. One of the major contributing factors to the increase of obesity worldwide is the inappropriate dietary intake and energy density of the diet, together with lower physical activity levels [1]. Given the high impact of nutrition on the development of the obesity epidemic, several urgent steps are needed to increase public awareness on this area and to encourage policy makers to combat this problem. Improving food quality, promoting healthy food at reasonable prices, mandatory food labelling, and protecting children and teenagers from negative food marketing are some examples of the needed actions.

This special issue focuses on recent research findings describing the effect of dietary and nutritional factors on the control of body weight and how would these findings be utilized towards the prevention and management of overweight and obesity.

Nutritional experience in the beginning of development can lead to epigenetic changes in the genome that influence the risk of obesity in later life. This epigenetic memory that persists into adulthood may have a role in the development of obesity and could be a potential reason for metabolic diseases later in life [2].

From epidemiological point of view, nutritional research in the area of obesity faces some challenges. These include choosing the optimal dietary assessment method, obtaining accurate and reproducible data, performing the proper data analysis, and drawing accurate conclusions. Dietary assessment relies on the ability of individuals to recall a complex collection of exposures and hence is associated with measurement error [3]. To overcome this issue, investigators do validate assessment methods against each other. However, the lack of objective reference biomarkers for most dietary exposures leaves us to rely on imperfect assessment tools. In line with the themes of the current special issue, five important and relevant articles are published.

Dietary approaches for improving overweight and obesity are the focus of X. Guo et al. who report the result of a 12-week randomized controlled intervention study in a Chinese population with overweight and obesity. By studying the effects of a meal replacement with 388 kcal in total energy at dinner time, the authors report improvement of body composition and metabolic parameters in the study subjects. Interestingly, the glucose and blood pressure reduction demonstrated in the study subjects is gender-specific, leading the authors to emphasize on the importance of gender stratification when a nutritional intervention study is designed for improvement of health.

The current issue's themes of “nutrients content and energy density in foods and beverages” and of “the dietary assessment methods in clinical studies” are investigated by H. Takase et al. who are describing their development of a dietary factor assessment tool for evaluating associations between visceral fat accumulation and major nutrients in Japanese adults. Their reported “tool” overcomes the need to actually measure the visceral fat mass which would not be feasible in large-population studies. By conducting dietary surveys and visceral fat measurement and analyzing the obtained data, the authors present the effect of appetite on visceral fat accumulation and on energy intake mainly from carbohydrate. In addition, they report the healthy food choice relation to visceral fat accumulation and to the dietary protein/fat ratio, dietary fiber/carbohydrate ratio, and N-3 fatty acid/fat ratio. Albeit being a large-population study, the authors propose its applicability at an individual level. Under the same theme, but conducting a different type of “cross-sectional” study, Charvet A. and F. G. Huffman describe their study entitled “Beverage intake and its effect on body weight status among WIC preschool-age children.” The intake of sugar-sweetened beverages is positively correlated with both the intakes of 100% fruit juice and milk, whereas water intake is negatively correlated. This study emphasizes on the need to limit 100% fruit juice intake and encourage water intake, which is important in order to reduce the risk for overweight and obesity in preschool-age children.

Investigating the cultural base underlying mothers' choice for their children milk is another article in this special issue and is submitted by L. P. Kim and N. Mallo. By combining and interpreting phone surveys and one-on-one interviews, the researchers are able to examine the association between maternal perceptions of self-weight and child-weight status and milk consumption behavior. The researchers report that perceiving the child to be overweight by his/her mother will guide her to make a healthier food choice for the family, namely, choosing reduced-fat milk instead of whole milk. The study emphasizes on the importance of considering the mothers' culturally driven perceptions and the pediatrician's influence when designing obesity prevention strategies for Hispanic families.

A further interesting article assessing different dietary and lifestyle factors as predictors of successful weight loss maintenance after follow-up for an 18-month period postbariatric surgery is presented by Masood A. and colleagues. The authors demonstrate that the patients' poor compliance to healthy dietary and behavioural lifestyle practices is the main cause for weight regain in this critical postbariatric period. This has led the authors to recommend more emphasis on the follow-ups and nutritional counseling sessions provided to the postbariatric patients to ensure maintaining the achieved beneficial weight loss after the surgery.
